# Non-alcoholic fatty liver disease: Role of PNPLA3 and its association with environmental chemicals

**DOI:** 10.46439/toxicology.6.029

**Published:** 2024

**Authors:** Shakil A Saghir, Ata Abbas, Saleh Alfuraih, Ajay Sharma, Jean Latimer, Yadollah Omidi, Rais Ansari

**Affiliations:** 1 ToxInternational Inc., Hilliard, OH, USA; 2 Department of Biological & Biomedical Sciences, Aga Khan Univ, Karachi, Pakistan; 3 Institute of Environmental Science and Meteorology, University of the Philippines-Diliman, Quezon City 1101 Philippines; 4 Division of Hematology and Oncology, Department of Medicine, Case Western Reserve University, Cleveland, OH 44106, USA; 5 Department of Pharmacology and Toxicology, Faculty of Pharmacy, Northern Border University, Rafha, Saudi Arabia; 6 Department of Pharmaceutical Sciences, Barry and Judy Silverman College of Pharmacy, Health Professions Division, Nova Southeastern University, Fort Lauderdale, FL, USA; 7 Department of Biomedical and Pharmaceutical Sciences, Chapman University School of Pharmacy, Chapman University, Irvine, CA, USA; 8 AutoNation Institute for Breast Cancer Research & Care, Nova Southeastern University, Fort Lauderdale, FL, USA

**Keywords:** Non-alcoholic fatty liver disease, Non-alcoholic steatohepatitis, Patatin-like phospholipase domain-containing protein 3, Liver fibrosis, Hepatocellular carcinoma

## Abstract

Globally, non-alcoholic fatty liver disease (NAFLD) is on the rise with 30–32%, 27–33%, 35–48%, 36%, 9–20%, and 36–38% prevalence in Asia, Europe, North America, South America, Africa, and Australia, respectively. Approximately, 5–10% of NAFLD proceeds to hepatitis called non-alcoholic steatohepatitis (NASH). NASH often progresses to liver fibrosis, cirrhosis, and hepatocellular carcinoma (HCC). Precise mechanism(s) for the development of HCC is not fully understood in NAFLD and NASH patients. Higher insulin levels in type 2 diabetes (T2D) can result in lipolysis of adipose tissue activating lipid synthesizing enzymes such as fatty acid synthase and stearoyl-CoA desaturase-1, resulting in lipid accumulation in liver. Higher levels of glucose in T2D patients activate carbohydrate response element binding protein and insulin which increases the level of active sterol regulatory element binding protein. These lipogenic transcription factors activate patatin-like phospholipase domain-containing protein-3 (PNPLA3) from their response elements in the promoter. Endocrine disrupting chemicals (EDCs) and persistent organic pollutants (POPs) are implicated in T2D development and NAFLD. The emerging association between POPs, including insecticides, fungicides, herbicides, and diabetes has been noted. However, their connection with NAFLD remains less evident. Here, we reviewed association of POPs, especially EDCs, and role of PNPLA3 in the development of NAFLD and NASH. We also reviewed the role of single nucleotide polymorphisms of PNPLA3’s association with NAFLD.

## Introduction

Non-alcoholic fatty liver disease (NAFLD) represents an escalating global health concern. Its prevalence varies significantly across different regions, in Asia it ranges from 30–32%, with notably higher prevalence in the Middle East. Europe reports a prevalence of 27–33%, while North America shows a range of 35–48%. In South America, prevalence is 36–59%, prevalence of 59% is from the meta-analysis of hospital-based studies that included patients with high risk for NAFLD and may not represent prevalence in the general population. Africa has the lowest reported prevalence of 9–20%; however, notable exceptions include Egypt with 57% and Sudan with 28% prevalence. Prevalence of NAFLD in Australia is 36–38% [1,2]. NAFLD is defined by lipid deposition exceeding 5% of liver weight in the absence of excessive alcohol consumption. NAFLD often progresses to non-alcoholic steatohepatitis (NASH) in many patients. NASH is an advanced stage of NAFLD, and it differs from alcohol-mediated steatohepatitis, which resolves with the cessation of alcohol usage. Whereas NASH can progress to fibrosis following hepatocyte death or cirrhosis; it even can lead to hepatocellular carcinoma (HCC) [3,4]. The hallmark of NAFLD is the mark accumulation of triglycerides in the liver, especially in hepatocytes. The sources of fatty acids for excessive liver triglycerides are adipose tissue lipolysis and increased *de novo* synthesis of lipids in liver [5,6]. Obesity (excessive food intake) and type 2 diabetes (T2D)-induced high blood glucose further worsen lipid levels in the liver [ 7–9].

**Figure 1.**
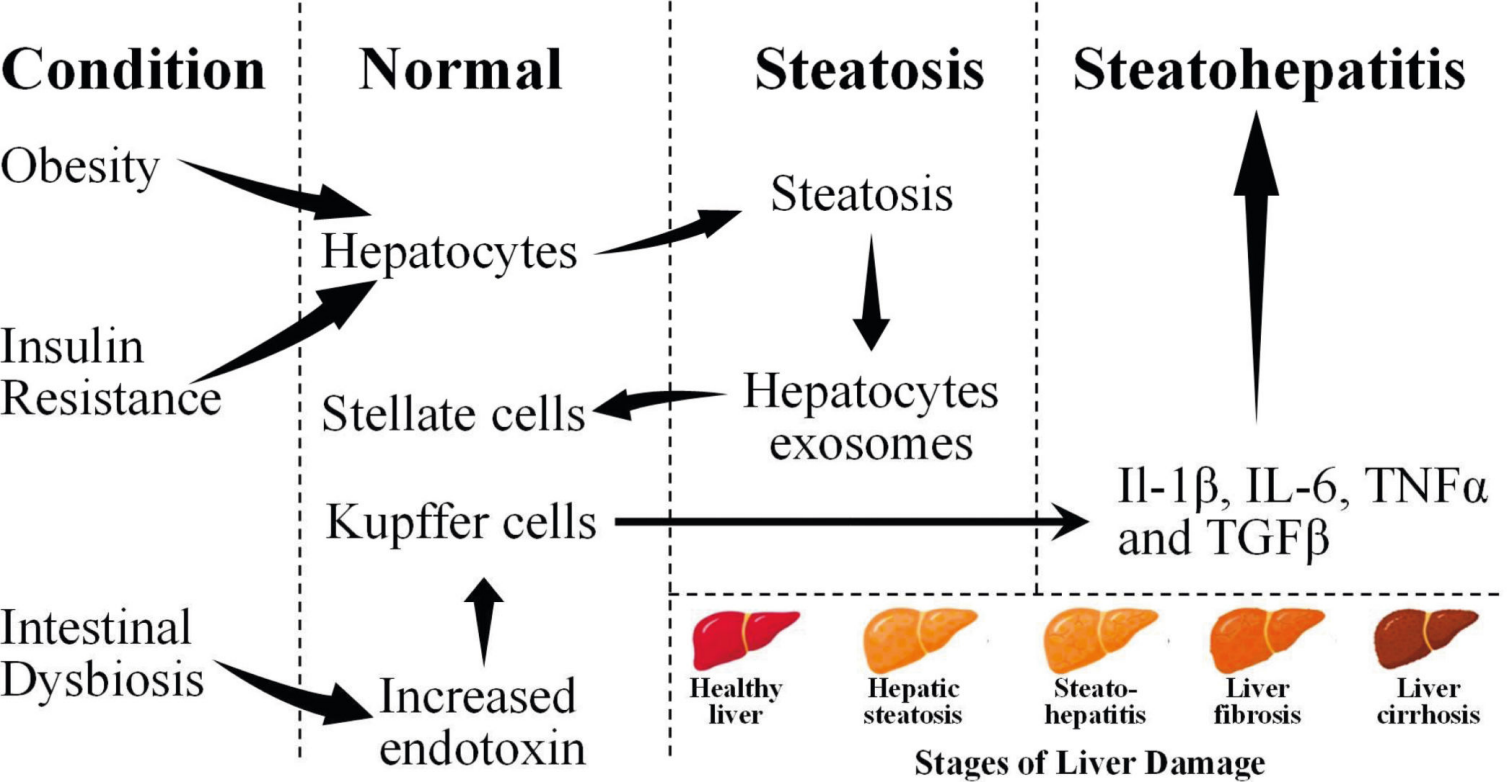
The conditions and transformation of normal liver to NAFLD, and NASH.

Obesity, T2D, and high-fat diet leads to fat accumulation in liver causing metabolic stress and predispose hepatocytes to apoptosis[10,11].The hepatocytes’ death activates hepatic stellate cells (HSCs) which secrete *alpha*-*smooth muscle actin (α-*SMA) that causes fibrosis of the liver[12,13]. Among many NAFLD patients, alterations in gut microbiota composition and function (gut dysbiosis) have also been observed. Gut dysbiosis results in increased permeability of intestinal membranes to bacterial-derived lipopolysaccharides which activates immune cells [14,15].In addition to the activation of immune cells, liver inherent immune, Kupffer cells, are also activated, leading to the progression of NAFLD to NASH [16]. On the other hand, activation of HSCs can trigger the secretion of TGF-β causing fibrosis and cirrhosis [17]. Additional impacts of the cirrhotic liver on HCCs are yet to be fully elucidated [18]. The process of NAFLD leading to HCC is summarized in Figure 1.

Among NAFLD patients, several gene variants appear to be associated with the disease. The genetic components associated with NAFLD include single nucleotide polymorphisms (SNPs) among the genes involved in the regulation of hepatic lipid turnover. The gene products of patatin-like phospholipase domain-containing 3 (PNPLA3), transmembrane 6 superfamily member 2 (TM6SF2), membrane-bound o-acyltransferase domain-containing 7 (MBOAT7), and glucokinase regulator (GCKR) have been found to associate with NAFLD [19]. Here, we present a comprehensive overview of the genes implicated in hepatic lipid accumulation, with specific focus on the PNPLA3 gene. We explore transcriptional regulation of PNPLA3 in the context of metabolic syndrome and T2D. The review underscores the pivotal role of genetic factors in the progression of NAFLD to NASH. We discussed the impact of an SNP in both the promoter and coding regions of PNPLA3, exploring how these polymorphisms influence the pathogenesis of NAFLD and NASH.

### Role of PNPLA3 Gene in Hepatosteatosis

PNPLA3 was cloned in 2001 from mouse 3T3 preadipocytes, which was a feeding-inducible gene and hence named adiponutrin [20] It is also known as calcium-independent phospholipase A2-epsilon (IPLA2epsilon) and chromosome 22 open reading frame20 (C22 or f20) [20,21]. PNPLA family contains 9 members (PNPLA 1–9), all members of the PNPLA family possess the patatin-like phospholipase domain (PNPLA1–9) [22,23]. Human PNPLA3 contains 9 exons and codes for 481 amino acids. It is localized on chromosome 22 (22q13.31) [23]. Human PNPLA3 gene is bigger (481 AA) than mouse gene (384 AAs) [23]. High expression of the human PNPLA3 gene has been observed in the liver while moderate expression is found in the brain, kidney, skin, and adipose tissues [22,24]. Human PNPLA3 possesses a single nucleotide polymorphism (rs738409) which changes the amino acid, isoleucine to methionine at 148^th^ position (I148M). A strong association was observed between the 148M variant (methionine at 148^th^ position) of PNPLA3 and hepatic fat levels in a genome-wide association study (GWAS) among Hispanic, African American, and European American individuals [25]. Additionally, multiple studies have found a strong association between liver cirrhosis and PNPLA3 148M variant [26–28]. This variant is also associated with alcoholic liver disease which proceeds from hepatosteatosis to steatohepatitis [29,30]. The variant 148M is implicated in the development of fatty liver in a transgenic mouse model of NAFLD [31]. The role of PNPLA3 is controversial [32–34]. The knockout of the PNPLA3 gene in mice results in neither fatty liver nor abnormal plasma and hepatic triglyceride level [35,36]. However, the knockin of human PNPLA3/148M in mice causes hepatosteatosis after sucrose feeding, and similarly, inflammation after feeding NASH diet [32–34].

The purified PNPLA3 protein, when expressed by baculovirus in Sf9 cells, has shown to possess triacylglycerol lipase and acylglycerol transacylase activities [21]. Huang et al. [37], when used Sf9-cell-expressed purified PNPLA3 protein, observed only lipase activity against triacylglyceride, diacylglyceride, and monoacylglyceride while transacylase activity remained absent. PNPLA3-expressed in lower eukaryotes, such as *Saccharomyces cerevisiae,* a yeast expression system, produced triglycerides hydrolase and retinyl esterase when retinyl-palmitate was used as a substrate [38,39 ]. Mutant 148M PNPLA3, when expressed in yeast, was substantially reduced, or completely lost the activity of triglyceride hydrolase [38,39]. Once expressed in mammalian cells (e.g., human embryonic kidney HEK293 cells), the purified human PNPLA3 displayed lipase activity [40]. The human PNPLA3 variant (148M), when expressed in Sf9 cells using baculovirus, was shown to lose triglyceride hydrolase activity when triolein was used as substrate [41].

The role of PNPLA3 in lipid metabolism is not well defined. This protein consistently associates with lipid droplets in mammalian cells [41–43]. Notably, both the wild-type PNPLA3 and its variant, 148M, exhibit abnormal, heightened associations with lipid droplets. This interaction disrupts lipid metabolism, leading to elevated lipid levels within mammalian cells. Furthermore, the degradation of the 148M variant, whether through ubiquitination or autophagy-related processes, is diminished. In contrast, the wild-type PNPLA3 undergoes normal turnover during feeding and fasting cycles [32,44,45]. It is presumed that the activity of another homolog, PNPLA2, which is also referred to as adipose triglyceride lipase (ATGL), is inhibited because the activator protein and comparative gene identification 58 (CGI-58), also referred to as abhydrolase domain containing 5 (ABHD5), can no longer competitively associate with PNPLA2, but associates at higher levels with mutant PNPLA3 [43,46,47]. PNPLA3 fails to localize to lipid droplets in CGI-58 liver knock-out cells, thus CGI-58 is needed to direct PNPLA3 association with lipid droplets [43].

There is one rare variant of PNPLA3 (i.e., rs2294918) in which amino acid glutamic acid (E) is changed to lysine (K) at 434 (E434K). The E434K variant attenuates the I148M mediated impact on steatosis and blood enzyme levels of liver injury markers, like aspartate transaminase (AST) and alanine transaminase (ALT) [48,49].

### Regulation of the PNPLA3 Gene in T2D and Metabolic Syndrome

**Figure 2.**
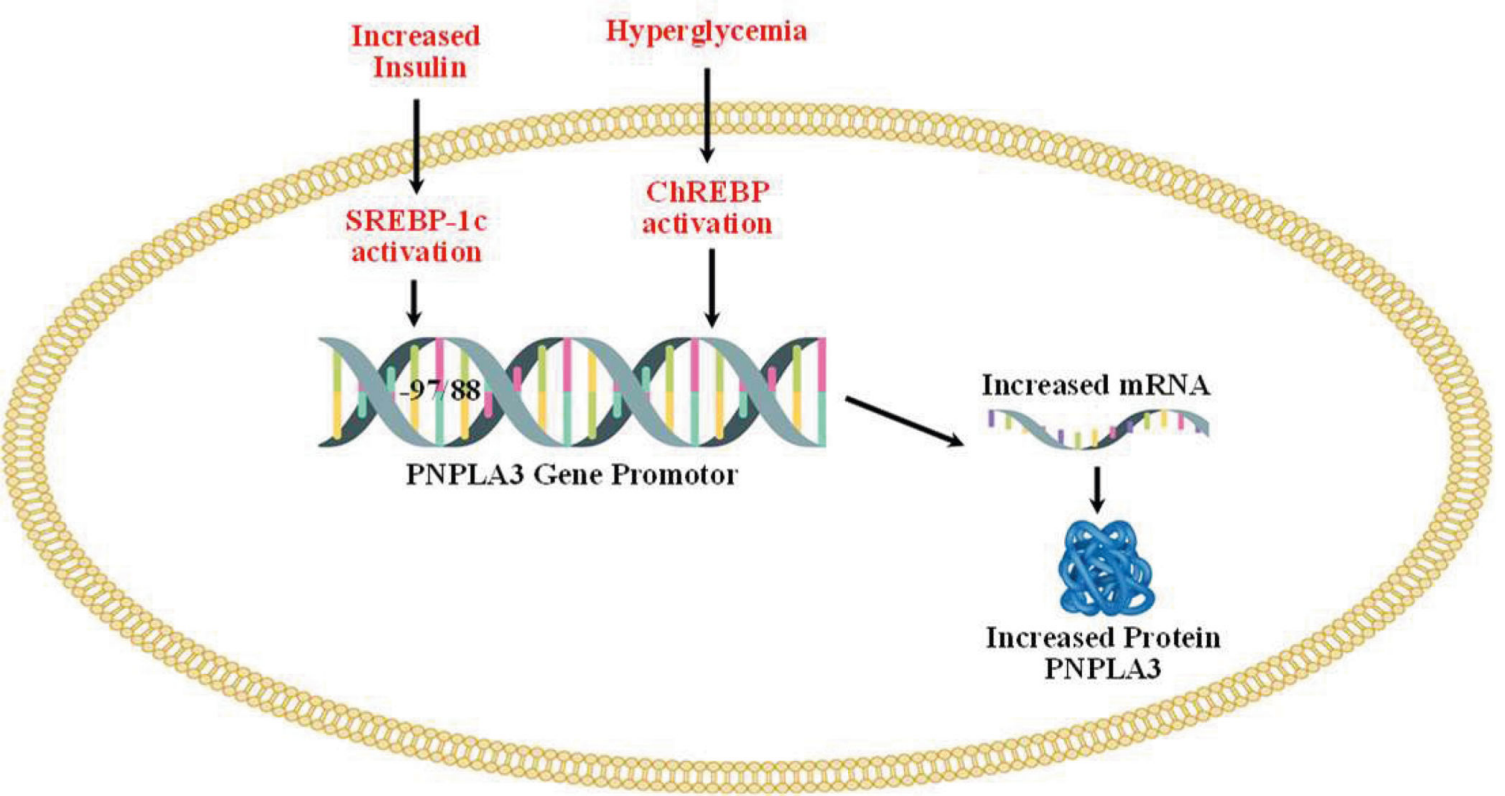
Regulation of the PNPLA3 gene by carbohydrate-response element binding protein (ChREBP) and sterol regulatory element binding protein-1c (SREBP-1c).

Regulation of the PNPLA3 gene by carbohydrate-response element binding protein (ChREBP) and sterol regulatory element binding protein-1c (SREBP-1c) are shown in Figure 2 below.

Both, ChREBP and SREBP-1c regulate PNPLA3 in human hepatocytes and in mouse liver [50–52]. SREBP-1c levels are increased in T2D because of increased levels of circulating insulin [53]. The binding of SREBP-1c to the promoter of PNPLA3 is increased by insulin treatment. Treatment with the inhibitor of PI3K (LY 294002) reduces the insulin-mediated promoter activity of PNPLA3 and SREB-1c expression in HepG2 cells. The response element of SREBP-1c (SRE) is located at −97/−88 from the start site [51]. SREBP-1c cooperates with the NFY transcription factor to increase the expression of PNPLA3 [51]. Overexpression of SREBP-1c in HepG2 cells increases the expression of PNPLA3 [51]. ChREBP is activated by higher blood glucose levels [54]. ChREBP transcriptional activity is regulated by intermediate products of glycolysis (glucose-6-phosphate (G-6-P) and fructose 2,6-bisphosphate), pentose phosphate pathway (xylulose-5-phosphate), and acetylation which is a posttranscriptional modification mediated by cyclic-AMP-response element binding (CREB) protein p300 [55–60]. ChREBP has two isoforms (i.e., ChREBPα and ChREBPβ), and in normal physiological conditions, ChREBPα is localized in the cytosol and upon glucose stimulation, localized to the nucleus and induces transcription of ChREBPβ, thus linking both isoforms in the regulation of activity. ChREBPβ remains localized in the nucleus that lacks glucose inhibitory domain (LID) which is present in ChREBP and thus ChREBP possesses more potent transcriptional activity [61,62]. Under low glucose levels, LID-mediated inhibition of ChREBPα occurs while ChREBPβ remains constitutively active [62]. ChREBP is involved in *de novo* lipogenesis and deletion of ChREBP genes decreases liver triglycerides level via inhibition of *de novo* lipogenesis [63]. Knockout of ChREBP on ob/ob mice reduced hepatic triglyceride levels [64]. Liver-specific silencing of ChREBP using an adenoviral-based silencing expression system reduced hepatosteatosis in ob/ob mice [65]. PNPLA3 remains in direct control of ChREBP and SREBP-1c in both mouse and human hepatocytes. Infection of adenoviruses expressing ChREBP and SREBP-1c to mouse and human hepatocytes increases the expression of PNPLA3 [50]. Thus, both these transcription factors increase the expression of PNPLA3 [50].

ChREBP is also activated by the liver X receptor (LXR) which heterodimerizes with retinoid X receptors and binds to the LXR response element. Two LXR binding elements are present at 2.4-kb upstream (+1) of the ChREBPα promoter, thus inducing the expression of the ChREBP gene, where ChREBPα can activate ChREBPβ as mentioned above [66]. The site is a response element of the nuclear hormone receptor superfamily which can also bind to thyroid receptors (TR); however, only TRβ but not TRα can activate ChREBP in the liver and white adipose tissues [67]. There is also an indirect link of PNPLA3 mutant with the fibrogenesis of the liver. Retinoic acids (all-trans) activate the retinoic acid receptor (RAR) and retinoid X receptor (RXR) transcription factors which downregulate fibrotic genes in HSCs [68–70] The variant PNPLA3 (148M) decreases retinol levels causing downregulation of RAR/RXR target genes in the hepatic stellate cell line [71].

### Role of Xenobiotics and Endocrine Disrupting Chemicals (EDCs) in Type 2 Diabetes

To address the growing needs of expanding human population and enhance overall health, it is essential to develop safe industrial, consumer, and agricultural products. Humans have synthesized a range of chemicals and introduced them into the environment to achieve several objectives. These synthetic chemicals, which do not naturally occur, serve diverse functions, including roles as pesticides, fertilizers, plasticizers, antimicrobials, detergents, fire-fighting agents like flame retardants, and others. Moreover, the production of various nanomaterials has been pursued, which may potentially influence genetics and epigenetics [72]. Notably, these chemicals, which can affect human and wildlife health via multiple pathways, have now become ubiquitous in the environment. Such contamination may result in different types of health issues, including cardiovascular disorders, pulmonary diseases, and disruption in the hormonal signaling pathways vital for endocrine functions. Such disruption in the hormonal signaling pathways, which is part of the endocrine system, is referred to as endocrine disrupting chemicals (EDCs) [73]. Table 1 describes the common chemicals which are used by humans and classified as EDCs and are also implicated in NAFLD [74]. The EDC list is ever increasing due to newer chemicals are continuously being introduced.

The identification of common structural characteristics among various chemicals that lead to ED is a significant challenge. These chemicals seem to induce ED through diverse mechanisms. Nevertheless, certain structural features might offer predictive insights regarding their potential as endocrine disruptors [75,76]. The harmful impacts of EDCs appear to associate with their capacity in terms of possible regulation of the transcription by the nuclear hormone receptor (NR) superfamily members and aryl hydrocarbon receptor [77–79] Different mechanisms of action might be involved by EDCs. For example, some of the xenobiotics, especially EDCs linked to T2D, are discussed below. Effects on T2D for the chemicals, primarily used in the production of various plastics, e.g., bisphenol A (BPA), appear to be controversial. Some studies have shown that BPA exposure might lead to possible insulin resistance, while possible *in-utero* exposure might result in long-term perinatal adverse effects [80]. Other studies have shown the association of BPA with the development of insulin resistance, loss of glucose homeostasis, and T2D among various populations [81]. The presence of BPA in urine has been reported to associate with the risk of T2D, while some other studies demonstrate no linkage between T2D and BPA exposure [80,82,83]. BPA exposure has shown to decrease the expression of adiponectin from adipocytes, while it seems to increase interleukin 6 (IL-6) and monocyte chemotactic protein 1 (MCP1) levels [84]. Hypoadiponectinemia was also shown to link to insulin resistance and T2D [85–88]. Likewise, urinary phthalates and their metabolites might associate with the initiation and/or progression of diabetes among women and geriatrics [89,90]. Of chlorinated hydrocarbons, chlordane and its derivatives found in pesticides might result in the exposure of humans via direct/indirect contact. Some studies have shown that oxychlordane, heptachlor, and chlordane exposure might associate with T2D [91] Exposure to parabens, which are alkyl esters of p-hydroxybenzoic acid, can simply happen as it has a wide antibacterial preservative application [92]. In a case-control study conducted in Jeddah, Kingdom of Saudi Arabia, parabens were found among individuals with T2D [93]. Further, the exposure of humans to pesticides and persistent organic pollutants (POPs) (e.g., oxychlordane, polychlorinated dibenzodioxins, dibenzofurans, dichlorodiphenyldichloroethylene (DDE), polychlorinated biphenyls (PCBs), hexachlorobenzene (HCB), and hexachlorocyclohexane) appears to increase the risk of T2D [94–97]. Similarly, exposure to chemicals such as insecticides and herbicides (pyrethroids, carbamates, 2,4-D, and 2,4,5-T) is a known risk factor for T2D [98–101]. It should be noted that exposure to chemicals such as BPA during pregnancy might increase the incidence of gestational diabetes [102]. Besides, gestational exposure to EDC might result in some epigenetic alterations, which can potentially predispose humans to different kinds of health risks including T2D [103]. During the first trimester of pregnancy, exposure to some chemicals (e.g., organophosphate (OP), organochlorine (OC), and carbamate (CB)) can markedly enhance the risk of gestational diabetes [104–106]. Additionally, upon poisoning with OP and CB pesticides, glycosuria has been observed transiently in human patients [107]. PCB, TCDD, *p’,p’*-DDE, are suspected to contribute to T2D [108–112]. Several studies in animals (e.g., rats, fresh-water fish, and European eel) have shown a significant rise in blood glucose levels after expose to insecticides like carbofuran, dimethoate, and fenitrothion [113–115]. Similarly, exposure of diazinon to rats, and carbaryl and phorate to freshwater fish have resulted in hyperglycemia [116–118]. The acute and subchronic exposure of rats and American kestrels to malathion resulted in hyperglycemia [119–121]. Despite such reports, there are some contradictory observations with other OP (e.g., soman and VX), in which glycemic states were not reported [122]. Remarkably, exposure to azynphos methyl, malathion, and diazinon in mice and rats was shown to alter the after-meal rise in glucose levels [123,124]. Readers are directed to Karami-Mohajeri and Abdollahi for detailed information about pesticides’ effects on hyperglycemia [125].

### Link of Insecticides Exposure to Liver Steatosis and Inflammation

To date, pesticides have been commonly used to control pests and vector-borne diseases, which seems to be necessary for high agricultural output and mitigating the world food requirement [126,127]. In a recent report, exposure to DDT + permethrin (PMT) at higher doses (DDT-15/PMT-15 vs DDT-150/PMT-150) has been reported to significantly enhance liver steatosis when hepatocytes grown on a chip were analyzed. Metabolite and transcriptomic analyses revealed the effects of DDT and PMT mixture on the development of the hepatosteatosis pathway [128]. Pesticides were implicated in toxicant-associated steatohepatitis (TASH) and NALFD [129]. Pesticides, like chlordecone, atrazine, and paraquat are implicated in steatohepatitis [130]. TCDD and PCB exposure to animals causes lipid accumulation in the liver; nevertheless, human exposure to TCDD and PCBs is not likely linked to hepatosteatosis [130]. Roundup, herbicide glyphosate, which is used in lawn management, exposure results in the fatty liver; exposure to minute quantities of glyphosate to rats has shown to develop fatty liver disease [131,132].

The data analysis of OC pesticides and NAFLD in the National Health and Nutrition Examination Survey (NHANS) (2003–2004) indicated that oxychlordane insecticide is associated with NAFLD [133]. The *p’p’*-DDT metabolite, *p’p’*-DDE, and trans-nonachlor were also associated with NAFLD but a statistically significant association was not observed [133]. The relationship between metabolic syndrome in non-diabetic adults and their serum levels of POPs has been noted in the NHANES data from 1999 to 2022. Furthermore, the connection between metabolic syndrome and NAFLD is well-established, particularly in adults with diabetes [134]. In one study, Wahlang observed that the insecticide and metal exposure was associated with NAFLD biomarkers in the NHANS (2003–2004) [135]. The U.S. EPA database with animal studies, known as the Toxicological Reference Database (ToxRefDB), was designed by the National Center for Computational Toxicology and the U.S. EPA Office of Pesticide Programs which includes pesticide registration data of the past 30 years. ToxRefDB found association of 42 toxicants (474 animal studies) with fatty liver disease (TAFLD), same as NAFLD [136]. Most of these compounds were pesticides, which included fungicides, herbicides, insecticides, and miticides [136–138]. The association of past OC insecticides usage, which are still present in the environment, has been established with new cases of fatty liver disease [137]. A summary of the studies for POPs, especially EDCs, to diabetes and NAFLD is presented in Table 1.

### Targeting PNPLA3 for the Treatment of NAFLD

Looking at the prevalence of I148M variant in Hispanics and its role in NAFLD, the very first approach is to down-regulate the expression of I148M by microRNA or antisense RNA. A recent study reported that a triantennary N acetylgalactosamine (GalNAC3) conjugated antisense oligonucleotides (ASO) targeting PNPLA3 significantly reduces liver steatosis, inflammation, and fibrosis in a 148M knockin mouse model [33]. Reduction in liver lipid is also observed by adeno-associated virus (AAV)-mediated shRNA delivery to 148M knockin mice [44]. Targeting the mutant (I148M) should be preferred as the function of WT-PNPLA3 is not well defined. Targeting PNPLA3 (148M) protein for degradation can be beneficial. Applying proteolysis-targeting chimera (PROTAC)-mediated degradation of Halo-tagged PNPLA3 variant (148M) has exhibited a significant effect on lowering hepatic lipid levels [44]. Another approach should be directed to CGI-58, binding with WT-PNPLA3 but not with PNPLA3 (I148M), which could activate WT-PNPLA3 and offer beneficial effects in lowering the hepatic lipid levels [23]. Therefore, a molecule or a mixture that activates WT-PNPLA3 and possibly degrade PNPLA3 variant 148M is needed before humans face the burgeoning crisis of NAFLD. A summary of studies of association of chemicals, especially EDCs, to diabetes and NAFLD is summarized in Table 1.

The U.S. Food and Drug Administration approved a new medicine, resmetirom (Rezdiffra^™^, Madrigal Pharmaceuticals, Inc.) on March 14, 2024, for patients who are progressing from NASH to fibrosis [177]. It is a highly selective thyroid hormone receptor-β (TRβ), a nuclear receptor superfamily, agonist. It significantly decreases intra-hepatic lipids by increasing mitochondrial β oxidation and thus improving hepatocyte mitochondrial function in later stage NASH patients [178].

## Concluding Remarks

The relationship between POPs (e.g., pesticides and EDCs) and the onset and progression of T2D is not yet fully understood, particularly for its connection to hepatic steatosis. Recent studies by the NHANES suggest a potential link between these chemicals and NAFLD. The growing prevalence of T2D globally underscores the significance of environmental factors, notably the impact of synthetic chemicals on metabolic disorders and their association with hepatosteatosis. Over the past thirty years, there has been a striking increase in the cases of diabetes and coincidingly escalating NAFLD incidences. The global prevalence of NAFLD among adults is estimated to be 32% (40% in males, 26% in females); prevalence has increased from 26% in or before 2005 to 38% in 2016 or later [179]. These trends indicate that while there may be various risk factors for these diseases, the exposure to POPs is increasingly being recognized as one of the critical factors contributing to the surge in diabetes and NAFLD cases.

Looking forward, it is imperative to deepen our understanding of how environmental pollutants like POPs interact with human health, particularly in the context of chronic diseases such as T2D and NAFLD. Such an understanding could lead to more effective strategies for prevention and management of these diseases. There is a growing need for rigorous research to explore the exact impacts of these chemicals on metabolic health holistically. Such studies would not only help in elucidating the mechanisms underlying the association between POPs and metabolic diseases but could also frame public health policies aimed at reducing exposure to these harmful substances. Furthermore, considering the escalating trends in T2D and NAFLD, there is an urgent requirement for public health initiatives that address the combined impact of lifestyle and environmental factors for these conditions. This holistic approach could be vital in mitigating the rising tide of these diseases in the coming decades and pave the way towards development of smart precision medicines for targeted therapy, such as the new FDA approved drug for these diseases. The new drug, resmetirom, is very recently approved by FDA for the treatment of patients who are progressing from NASH to fibrosis but not for the treatment/prevention of NAFLD. Fortunately, several clinical trials are underway (https://clinicaltrials.gov/) and we hope treatments for NAFLD will also become available soon.

## Figures and Tables

**Table 1.** A summary of studies related to association of POPs especially the EDCs to diabetes and NAFLD.

Chemicals	EDC	Diabetes	NAFLD
Hexachlorocyclohexane	139	+ 140	+ 141
Chlordecone	142		+ 143
Polychlorinated Biphenyls (PCBs)/Aroclor, polychlorinated dibenzofuran (PCDFs)	+ 139, 144, 145	+ 139, 146	+ 147
Aldrin, chlordane. Heptachlor, dichlorvos, trichlorfon and cyanazine	145,148, 149, 150, 151 152	+ 153	
*trans*-nonachlor, oxychlordane, β-hexachlorocyclohexane, *p,p*’-DDT, and *p,p*’-DDE	139	+ 154, 155	156
Oxychlordane, Chlordanes	+ 139	+ 149	+ 133
Pyrethroid	157	100	+ 128
2,4-dichlorophenoxy acetic acid (2,4-D) 2,4,5-T/2,4,5-TP	144	+ 158	
Fonofos, Phorate, parathion	139	+ 158, +159	
Pesticides including organochlorines, organophosphates, pyrethrins, and herbicides		153, 160, 161, 162	
Cyproconazole, Dazomet, Fluazinam, hexaconazole, Pyrasulfotole metabolite, and Acequinocyil			163, 136
Myclobutanil			+164
Mancozeb	165		+166
Hexaconazole	167		+ 168
Glyphosate	169	+ 170	+131, 132, 170
PFAS	139		+171
PFOA, PFOS	172		+173, 174

+ shows positive impacts. For details, readers are directed to 73, 74, 138, 175, 176.

## Highlights

- Globally, NAFLD is on the rise - prevalence is ~32% (40% in males, 26% in females) which increased from 26% to 38% between ≤ 2005 and ≥ 2016.
- Prevalence is 30–32%, 27–33%, 35–48%, 36%, 9–20%, and 36–38% in Asia, Europe, North America, South America, Africa, and Australia, respectively.
- ~5–10% of NAFLD proceeds to NASH and liver fibrosis, cirrhosis, and HCC.
- Higher insulin in T2D can cause lipolysis activating lipid synthesizing enzymes and lipid accumulation by activating carbohydrate response element binding protein and insulin.
- Increase in active sterol regulatory element binding protein activate PNPLA3.
- EDCs and POPs are implicated in T2D development and NAFLD along with single nucleotide polymorphisms of PNPLA3.
